# Woodland caribou habitat selection patterns in relation to predation risk and forage abundance depend on reproductive state

**DOI:** 10.1002/ece3.4124

**Published:** 2018-05-04

**Authors:** Rebecca Viejou, Tal Avgar, Glen S. Brown, Brent R. Patterson, Doug E. B. Reid, Arthur R. Rodgers, Jennifer Shuter, Ian D. Thompson, John M. Fryxell

**Affiliations:** ^1^ Department of Integrative Biology University of Guelph Guelph ON Canada; ^2^ Ontario Ministry of Natural Resources and Forestry Sault Ste. Marie ON Canada; ^3^ Wildlife Research and Development Section Ontario Ministry of Natural Resources and Forestry Peterborough ON Canada; ^4^ Centre for Northern Forest Ecosystem Research Ontario Ministry of Natural Resources and Forestry Thunder Bay ON Canada; ^5^ Canadian Forest Service Sault Ste. Marie ON Canada

**Keywords:** caribou, food availability, habitat selection, predation risk, reproductive state, video collars

## Abstract

The ideal free distribution assumes that animals select habitats that are beneficial to their fitness. When the needs of dependent offspring differ from those of the parent, ideal habitat selection patterns could vary with the presence or absence of offspring. We test whether habitat selection depends on reproductive state due to top‐down or bottom‐up influences on the fitness of woodland caribou (*Rangifer tarandus caribou*), a threatened, wide‐ranging herbivore. We combined established methods of fitting resource and step selection functions derived from locations of collared animals in Ontario with newer techniques, including identifying calf status from video collar footage and seasonal habitat selection analysis through latent selection difference functions. We found that females with calves avoided predation risk and proximity to roads more strongly than females without calves within their seasonal ranges. At the local scale, females with calves avoided predation more strongly than females without calves. Females with calves increased predation avoidance but not selection for food availability upon calving, whereas females without calves increased selection for food availability across the same season. These behavioral responses suggest that habitat selection by woodland caribou is influenced by reproductive state, such that females with calves at heel use habitat selection to offset the increased vulnerability of their offspring to predation risk.

## 1 INTRODUCTION

Animals need to acquire energy to survive, grow, and reproduce. Mobile animals can increase their energetic intake by selecting habitats where more energy is available. The ideal free distribution predicts that all else being equal, animals in a landscape with heterogeneous resource availability should distribute themselves in proportion to the amount of resources available (Fretwell & Lucas, 1969). However, animals are not expected to use habitat selection to maximize energetic intake in the presence of an immediate threat to survival. For example, some prey species will select habitats with lower predation risk even if this constrains their access to energy (Hernández & Laundré, 2005). Prey species exposed to both top‐down and bottom‐up constraints on fitness need to balance the functional behaviors of food acquisition and predator avoidance (Brown, 1999).

Habitat selection is not a static behavior of an individual or population. Animals can have different habitat selection patterns across spatial scales (Johnson, 1980) and adjust what they select over time (e.g., Dardaillon, 1986; Sakuragi et al., 2003) with changing internal requirements and external environments (Jones & Boulding, 1999; Martin & Lopez, 1999). The internal requirements of the individual can also result in predictable differences in habitat selection between age classes, sexes, and reproductive states (McLellan & Shackleton, 1988). Testing for a relationship between reproductive state and habitat selection is particularly challenging for wide‐ranging animals in relatively inaccessible landscapes, as it is difficult to assess changes in reproductive state after an individual is collared (however, see Pinard, Dussault, Ouellet, Fortin, & Courtois, 2012).

In this study, we introduce a novel method of identifying the reproductive state of large mammals using animal‐borne video collars on woodland caribou (*Rangifer tarandus caribou*) in northern Ontario. A substantial body of evidence is accumulating that woodland caribou populations are declining worldwide (Vors & Boyce, 2009). Habitat alteration has been implicated as a contributory mechanism for the northward shift in the limit of the caribou range in Ontario (Vors, Schaefer, Pond, Rodgers, & Patterson, 2007) as well as declines in caribou populations elsewhere in Canada (Wittmer, McLellan, Serrouya, & Apps, 2007). One explanation for the decline is that a numerical response of moose (*Alces alces*) to regenerating forests in woodland caribou ranges (Potvin, Breton, & Courtois, 2005) has led to a numerical response in wolves (*Canis lupus*, Seip, 1992). Although moose are their primary prey, wolves also prey opportunistically on adult caribou (Seip, 1992) and might limit the woodland caribou population through apparent competition with moose (James, Boutin, Hebert, & Rippin, 2004).

There are good reasons to suspect that calf survival may also be limited by predation. Young calves are particularly vulnerable to predation (Adams, Singer, & Dale, 1995), and calf survival is higher when caribou are spatially segregated from wolves (Seip, 1992). In mountainous areas, females have been found to calve at high elevation despite reduced access to high‐quality forages, potentially to avoid predation (Bergerud, Butler, & Miller, 1984; Bergerud & Page, 1987). Although it has been observed that the majority of calf mortality is likely caused by predation rather than other or unknown causes (Pinard et al., 2012), reproductive caribou must also acquire sufficient food resources to rear a calf. Lactating females require twice the daily maintenance energy of nonlactating females (Chan‐McLeod, White, & Holleman, 1994), suggesting that energy might also limit calf survival.

Here, we test whether female woodland caribou in the boreal forest of northern Ontario select local habitats to reduce predation risk, enhance access to energy‐rich forage plants, or both. By comparing habitat selection of females with a calf to those without a calf, we also test whether females use habitat selection to meet the increased energetic demands of lactation, avoid the increased predation risk to their offspring, or both. We use previously defined landscape models of dietary digestible biomass to estimate food availability (Avgar et al., 2015) and relative wolf density to estimate predation risk (Kittle et al., 2015). We also consider the proximity of roads as an additional contributor to perceived predation risk.

We use two spatial analyses to compare habitat selection patterns, with available habitat defined by seasonal range in the first, and constrained by the movement capacity of caribou in the second. To complement our spatial analyses, we compared patterns in habitat use in precalving and postcalving seasons. We expect that if females with calves use habitat selection to meet increased energetic demands, they would select more strongly for food availability than females without calves. Similarly, if females with calves use habitat selection to account for the increased vulnerability of their offspring to predation, they would avoid predation risk more strongly than females without calves. In addition, females would use habitat with lower predation risk after calving compared to before calving. By testing these predictions, we aim to determine whether the presence of young calves affects the distribution of woodland caribou, identify what top‐down and bottom‐up influences on fitness might drive differences in habitat selection, and provide an example of the utility of video collars in wildlife ecology.

## 2 METHODS

### 2.1 Study area

We used data previously collected from a 142,172‐km^2^ region within the boreal forest of Ontario (from 49°32′to 52°45′N and 84°27′ to 93°23′W) that spans a wide gradient in caribou fitness attributes. The levels of human disturbance were relatively low in the northwestern end of the study landscape (centered on the township of Pickle Lake) because commercial forestry operations were not permitted, and relatively high in the southeastern end of the study landscape (centered on the township of Nakina) due to commercial timber harvesting. Pickle Lake accordingly has a higher proportion of old conifer stands and lower moose, wolf, and road densities compared to Nakina, where mixedwood and deciduous stands are more common (Mallon, Turetsky, Thompson, Fryxell, & Wiebe, 2016).

### 2.2 Animal data collection

We used video and telemetry data previously collected from 19 caribou captured near the Pickle Lake and Nakina townships between 2011 and 2012. The caribou were net‐gunned from a helicopter and fitted with Lotek GPS and Argos camera collars (Thompson et al., 2012), in accordance with approved Ontario Ministry of Natural Resources and Forestry animal care protocols (11‐183 and 12‐183). The cameras recorded 10‐s video clips every 10 min during daylight hours, while telemetry points were obtained every 2.5 hr. GPS fixes that implied unreasonable movement rates were removed from the data set (Avgar et al., 2015). We only included caribou with videos recorded during the spring and summer, when calves are young. This resulted in a subset of nine individuals, of which five were closer to the Nakina end of the study area and four near the Pickle Lake end. We identified reproductive state from the video recordings, by assuming individual caribou were accompanied by their calves on all days between the first and last calf sightings in the videos, and without calves otherwise.

### 2.3 Landscape covariates

To quantify food availability and predation risk, we used two landscape models (of dietary digestible biomass and relative wolf density, respectively) that were developed during previous research in our study area (Avgar et al., 2015; Kittle et al., 2015). The landscape models defined habitats by dividing the landscape into a hexagonal grid (i.e., resource units), with 500 m between cell centers (cell area = 0.22 km^2^), in ArcGIS 10.1.

To estimate dietary digestible biomass (kg/m^2^), Avgar et al. (2015) used plant biomass samples from 162 forest stands collect in the summers of 2010–2012 (Mallon et al., 2016). These measurements were converted to digestible biomass and weighted by caribou summer (16 June–31 October) diet composition (Newmaster et al., 2013; Thompson et al., 2015). Dietary digestible biomass values were projected across the landscape based on their statistical relationship with relative elevation, the Normalized Difference Vegetation Index (NDVI; NASA LP DAAC 2013), and Far North Land Cover database (FNLC v1.3.1; Ontario Ministry of Natural Resources 2013). This resulted in a static model of summer dietary digestible biomass (see Avgar et al., 2015 for details).

To estimate relative wolf density, Kittle et al. (2015) fit 95% Brownian bridge utilization kernels for 34 packs using telemetry data from 52 wolves in the study area recorded during the winters (November–April) of 2010–2011 and 2011–2012. Using equivalent methods, Avgar et al. (2015) extended these models to the summers (May–October) of 2010 and 2011. The fix rate was 2.5 hr the first summer and 5 hr the second. These values were normalized by dividing each value by the sum of all values and then weighed by the number of individuals in a pack. The pack utilization distributions were added together to estimate a population‐level utilization distribution. Relative wolf density was then projected across the landscape (maintaining the hexagonal grid) by linking the population utilization distribution to relevant landscape covariates (NDVI, FNLC land cover type, and proximity to roads, dumps, towns, and waterways) using generalized least squares regression models (see Kittle et al., 2015 for details). This resulted in estimates of relative wolf density (predation risk) that are reflective of the habitat that wolves in our study area tend to occupy in general during the summer, rather than the habitat selection patterns of specific packs.

We also accounted for the effect of proximity to roads on habitat selection. Caribou tend to avoid roads due to human activity (Dyer, O'Neill, Wasel, & Boutin, 2001) and increased predation risk (Whittington et al., 2011). Although the effect of roads on predator density was already accounted for in the predation risk model, human activity on and around roads can act as a particularly conspicuous cue for risk, and caribou might avoid them more efficiently than other landscape features that are associated with predator density. Distance (in kilometers) was calculated from the center of each hexagonal cell to the nearest paved, primary, or secondary road, or rail line, using road locations and classifications provided by the Centre for Northern Forest Ecosystem Research, Ontario Ministry of Natural Resources and Forestry. Distances were inverted, resulting in a measurement of proximity to roads. The three covariates have not all been standardized, so comparisons of the strengths of selection across covariates cannot be made in the final models.

### 2.4 Resource selection function

To determine whether caribou habitat selection depends on reproductive state at the seasonal‐range scale, we fit a resource selection function (RSF) of the used–available design described by Manly, McDonald, Thomas, McDonald, and Erikson (2002). A RSF is any function that is proportional to the probability of selection of a given habitat (Lele, Merrill, Keim, & Boyce, 2013; Manly et al., 2002). We defined the calving season as the period between the first and last calf sightings across all caribou (12 May–1 September), and only used fixes recorded during this period. We removed any fixes that were taken beyond the last recording date of the videos of each individual, as reproductive status could no longer be determined. For individuals that were observed with calves, we also removed fixes between the start of the calving season and the first calf sighting because we were interested in habitat selection while calves were at heel, rather than during gestation. The subsequent analysis required the exclusion of any fixes that were not the final of three fixes taken consecutively at 2.5‐hr intervals to calculate turn angles (see SSF below). We removed these fixes for the seasonal‐range‐scale analysis as well, so that the same subset of fixes defined used habitat for each spatial analysis. Finally, if the above criteria resulted in a sample size of 100 or fewer fixes per caribou per calf status, we also removed those fixes.

The estimates of RSFs depend on how available habitats are defined (Avgar, Potts, Lewis, & Boyce, 2016; Boyce et al., 2003; Johnson, 1980; Prokopenko, Boyce, & Avgar, 2017). For our coarse‐scale analysis, we considered the habitat selection of caribou within their seasonal range defined by 95% minimum convex polygons estimated from all fixes during the calving season, using the adehabitatHR package in R 3.0.1 (Calenge, 2006). Available locations were randomly drawn from each seasonal range at a ratio of 10 available locations for every used location.

We estimated the exponential‐RSF parameters using a mixed‐effects GLM with a binomial link function comparing the distributions of used and available habitats (Gillies et al., 2006; Manly et al., 2002). A random intercept per individual caribou was included to account for the lack of independence of observations from the same individual (Gillies et al., 2006; Hebblewhite & Merrill, 2008). This estimated RSFs of the form:

(1) $$w\left(x\right)=\exp\left(\beta_{0}+\beta_{1}x_{1ij}+\ldots +\beta_{n}x_{\mathrm{nij}}+\gamma_{0j}\right),$$

where *w*(*x*) is proportional to the probability of a given habitat being selected, the β_*n*_ coefficients are the strength of selection for *n* ^th^ covariate (predation risk, food availability, and proximity to roads), *x*_*nij*_ is the value of the *n* ^th^ covariate at the *i* ^th^ location of the *j* ^th^ individual, β_0_ is an arbitrary scaler, and γ_0*j*_ is the random intercept per individual. To determine whether females of a given reproductive state selected or avoided any of the landscape covariates, a RSF was run for each reproductive state separately (Appendix S1A). To determine whether habitat selection differed with the presence of a calf, a RSF including the interaction of each covariate with reproductive status (without calf = 0; with calf = 1) was run using all fixes. Because we had individuals from two study areas with known differences in human disturbance, we also ran the above RSF with interaction separately for each end of the study area. Each of these models was based on few individuals (five in Nakina and four in Pickle Lake), so while we are reluctant to draw conclusions from them, we did use them to check the consistency of our results across the study area (Appendix S1B). All coefficients were estimated using the lme4 package for R 3.0.1.

### 2.5 Step selection function

Although RSFs can reveal relevant habitat selection patterns at the seasonal‐range scale, there is an implicit assumption that the entirety of an individual's seasonal range is equivalently “available”, regardless of the animal's current location. Given that closer habitats are generally more accessible than distant ones, step selection functions (SSFs) have been developed to reduce reliance on the assumption of equivalent availability (Thurfjell, Ciuti, & Boyce, 2014). To determine whether caribou habitat selection depends on reproductive state when movement constraints are accounted for, we fit a step selection function (SSF) of the used–available design described by Fortin et al. (2005). SSFs apply realistic spatial constraints on what is available to an individual at observed locations by taking the movement patterns of all individuals in the population into account. A step is the Euclidean distance between two consecutive fixes, and used habitats were defined by the endpoint of every step. These endpoints were equivalent to the used fixes for the RSF analysis. Each triplet of three consecutive fixes was used to determine the step length and turn angle (angular deviation between the orientation of two consecutive steps) distributions of the nine individuals combined.

We randomly assigned ten available steps beginning at the start point of each used step. Endpoints for the ten available steps were defined by drawing pairs of turn angles and step lengths from the empirical distributions. Used and available steps were then paired by their shared start points for the case‐controlled logistic regression of used relative to available endpoints across food availability, predation risk, and proximity to roads. To determine whether females of a given reproductive state selected or avoided any of the landscape covariates, a SSF was run for each reproductive state separately (Appendix S1A). To determine whether habitat selection differed across reproductive state, a SSF including the interaction of each covariate (predation risk, food availability, and proximity to roads) with reproductive state (without calf = 0; with calf = 1) was run using all steps. As in the RSF, we also fit a separate SSF with interaction for the Pickle Lake and Nakina ends of the study area (Appendix S1B). All coefficients were estimated using the survival package for R 3.0.1.

### 2.6 Latent selection difference function

Habitat selection and movement by woodland caribou are known to vary seasonally with respect to food availability and predation risk (Avgar, Mosser, Brown, & Fryxell, 2013; McGreer et al., 2015). To compare habitat selection of caribou during the precalving and postcalving seasons, we used mixed‐effects logistic regression to estimate the coefficients of a latent selection difference (LSD) function (Fischer & Gates, 2005; Roever, Boyce, & Stenhouse, 2008). An LSD function contrasts the habitat use of two classes of locations. The two classes of concern for our analysis were locations in the precalving season and locations in the postcalving season. For individuals that were eventually observed with a calf (successful calving = 1), we defined the precalving season as 40 days prior to the first calf sighting of each individual, and the postcalving season as 40 days after. Calves are typically able to satisfy their own nutritional requirements 40–45 days after birth (Lavigueur & Barrette, 1992). For females that were never observed with calves (successful calving = 0), we used locations recorded 40 days before and after the median Julian date of the first calf sightings (Demars, Thiessen, & Boutin, 2011). We used mixed‐effects logistic regression of season (postcalving = 1; precalving = 0) across the three landscape covariates (food availability, predation risk, and proximity to roads) to estimate the LSD coefficients. By including the interaction of successful calving with each landscape covariate, we could differentiate seasonal changes in habitat selection that were independent of calving from changes associated with calving. The LSD function is of the form:

(2) $$p\left(x\right)=\exp\left(\beta_{0}+\beta_{1}x_{1ij}+\ldots +\beta_{n}x_{\mathrm{nij}}+\gamma_{0j}\right),$$

where *p*(*x*) is the probability of caribou selecting a given habitat postcalving relative to precalving, the β_*n*_ coefficients represent the relative difference in habitat use across seasons for each covariate, *x*_*n*_, β_0_ is an arbitrary scaler, and γ_0*j*_ is the random intercept per individual (Latham, Latham, & Boyce, 2011). Although relative change in habitat use is directly measured, inference can be extended to relative change in habitat selection if it is reasonable to assume that the same habitats are available across both seasons. We estimated a 95% MCP home range for the pre‐ and postcalving seasons of each individual to visually assess whether these areas were proximate enough to assume that the precalving areas remained available to the animals in the postcalving season (Appendix S1D).

## 3 RESULTS

For the spatial analyses (RSF and SSF), our final sample included nine individual caribou, of which three were observed with calves throughout the entire calving season, three were observed with calves for part of the calving season, and three were never observed with calves. The three that had a sufficient number of fixes (>100) both with and without a calf at heel contributed observations to both reproductive states. We therefore had a total of six individuals with a calf (3,662 fixes total) and six individuals without a calf (2,651 fixes total), accounting for the overlap of three individuals (Table 1). The average seasonal‐range size (±*SE*) in the RSF was 213.11 ± 93.90 km^2^, while the median step length in the SSF was 119.23 m (see Appendix S1C for step length and turn angle distributions). For the temporal analysis (LSD), we had six individuals that calved (2,323 fixes precalving and 2,302 fixes postcalving) and three individuals that did not (1,168 fixes precalving and 1,166 fixes postcalving). Because the number of days and fix rates were constant across individuals, the total number of fixes per individual in the seasonal analysis was relatively uniform (mean ± *SE* of 773 ± 8 fixes).

**Table 1:** Number of fixes per individual across reproductive state. The five individuals including “AU” in the animal ID were collared on the Nakina (Auden) side of the study area, whereas the four with “PL” were collared on the Pickle Lake side

Animal ID	Number of fixes	
	With calf	Without calf
CAU243	0	554
CAU247	922	0
CAU248	864	0
CAU264	490	199
CAU310	0	442
CPL205	0	455
CPL208	558	0
CPL320	542	493
CPL97168	306	508

### 3.1 Resource selection function

**Figure 1:**
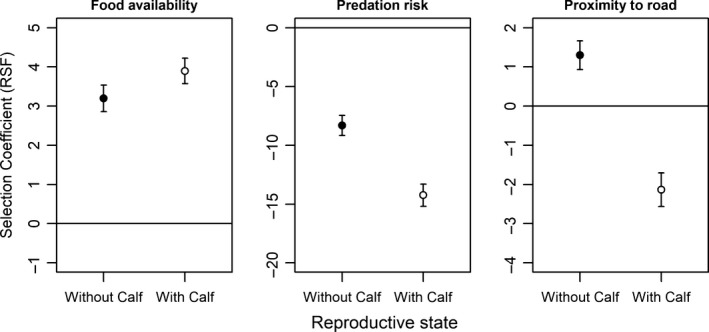
Resource selection function (RSF) coefficients for food availability (dietary digestible biomass), predation risk (relative wolf density), and proximity to roads. Coefficients were estimated in two separate models (with calves at heel = open circles; without calves = solid circles) using mixed‐effects logistic regression. The bars indicate 95% confidence intervals

The available locations drawn from caribou seasonal ranges had a mean ± *SE* of 0.18870 ± 0.00015 for relative wolf density and 0.18482 ± 0.00045 for dietary digestible biomass and were 14.349 ± 0.050 km from roads. The RSF showed that caribou used habitats associated with all three landscape variables disproportionately relative to what was available within individual calving ranges. Females avoided predation risk both with (β = −14.239; *p* < .0001) and without (β = −8.315; *p* < .0001) calves at heel (Figure 1; Appendix S1A), and females with calves did so more strongly than did females without a calf (β = −5.6858; *p* < .0001; Table 2). All females selected for food availability (β = 3.897 with calves; β = 3.200 without; *p* < .0001), and the strength of habitat selection was not significantly different across reproductive state (β = 0.3402; *p* = .1434; Table 2). While accounting for predation risk, females with a calf avoided roads (β = −2.131; *p* < .0001), whereas those without a calf selected for habitats near roads (β = 1.299; *p* < .0001), and this difference in selection across reproductive state was significant (β = −2.4386; *p* < .0001; Table 2). Although the selection strengths were different between the two ends of the study area (Pickle Lake and Nakina), the direction and significance of the effects of having a calf at heel (the estimates for the interaction terms) were consistent, with the exception that in Pickle Lake, females with calves selected forage more strongly than those without, while in Nakina, females with calves selected forage less strongly than those without (Appendix S1B).

**Table 2:** Resource selection function (RSF) of caribou habitat selection for predation risk (PRED), food availability (FOOD), and proximity to roads (ROAD) accounting for the presence/absence of a calf (CALF; without calf = 0, with calf = 1). Main effects estimate selection strength by females without calves, and interaction terms estimate the additional effect on selection strength from having a calf at heel (* indicates significance at a = 0.05)

Covariate	β	*SE*	*z*	*p*
Intercept	−1.4670	0.1058	−13.862	<.0001*
PRED	−8.5657	0.4456	−19.221	<.0001*
FOOD	3.4163	0.1730	19.221	<.0001*
ROAD	0.7000	0.2009	3.484	.000494*
CALF	1.2008	0.1298	9.254	<.0001*
PRED x CALF	−5.6858	0.6558	−8.670	<.0001*
FOOD x CALF	0.3402	0.2325	1.463	.143437
ROAD x CALF	−2.4386	0.2601	−9.375	<.0001*

### 3.2 Step selection function

**Figure 2:**
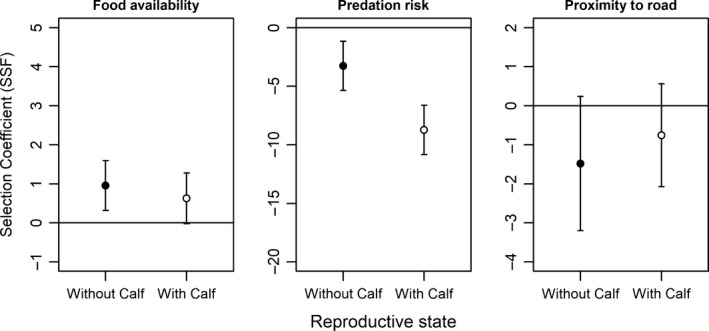
Step selection function (SSF) coefficients for dietary digestible biomass (dietary digestible biomass), predation risk (relative wolf density), and proximity to roads. Coefficients were estimated in two separate models (with calves at heel = open circles; without calves = solid circles) using case‐controlled logistic regression. The bars indicate 95% confidence intervals

The available locations drawn using empirical step length and turn angle distributions had a mean ± *SE* of 0.17350 ± 0.00011 for relative wolf density and 0.21868 ± 0.00041 for dietary digestible biomass and were 13.831 ± 0.044 km from roads. The SSF indicated that all females used habitat associated with low predation risk disproportionately relative to what was available within a 2.5‐hr step (median step length = 119.23 m) from each observed location (Figure 2; Appendix S1A). Predation risk was avoided by females with a calf (β = −8.732; *p* < .0001) as well as those without calves (β = −3.262; *p* = 0.0023; Figure 2), but females with a calf avoided predation risk more strongly (β = −5.470; *p* = .0003; Table 3). Females without calves selected for habitats with high levels of dietary digestible biomass (β = 0.955; *p* = .0034; Figure 2), whereas those with a calf did not. Roads were not selected or avoided. The selection strengths were different between the two ends of the study area (Pickle Lake and Nakina), but the direction and significance of the effects of having a calf at heel were consistent with the exception that females with calves in Pickle Lake had significantly weaker selection for food availability than females without calves in the same area (β = −1.649; *p* = .0263; Appendix S1B).

**Table 3:** Step selection function (SSF) of caribou habitat selection for predation risk (PRED), food availability (FOOD), and proximity to roads (ROAD) accounting for the presence/absence of a calf (CALF; without calf = 0, with calf = 1). Main effects estimate selection strength by females without calves, and interaction terms estimate the additional effect on selection strength from having a calf at heel (* indicates significance at a = 0.05)

Covariate	β	*SE*	*t*	*p*
PRED	−3.2617	1.0685	−3.052	.002270*
FOOD	0.9553	0.3262	2.929	.003403*
ROAD	−1.4811	0.8784	−1.686	.091767
PRED x CALF	−5.4704	1.5154	−3.610	.000306*
FOOD x CALF	−0.3272	0.4647	−0.704	.481374
ROAD x CALF	0.7238	1.1056	0.655	.512659

### 3.3 Latent selection difference function

Habitat selection of all females changed from precalving to postcalving seasons. Females without calves used locations with lower predation risk (β = −7.089; *p* < .0001) and higher food availability (β = 8.516; *p* < .0001) and closer to roads (β = 1.233; *p* = .0277) during the 40 days following the median calving date relative to the 40 days prior (Table 4). Females with a calf at heel lowered use of areas with high predation risk to a greater degree upon calving (β = −19.234; *p* < .0001) compared to seasonal changes in habitat use of females without calves (Table 4). Females with a calf reversed the increased use of food availability observed between the pre‐ and postcalving seasons of females without calves (β = −7.995; *p* < .0001; Table 4). The seasonal selection difference for proximity to roads was lower for females with calves than for those without calves (β = −9.865; *p* < .0001; Table 4). Of the nine individuals, eight had overlapping 95% MCP home range estimations in the pre‐ and postcalving seasons (Appendix S1D). The minimum distance between the two seasonal ranges for the individual without overlap (CPL320) was 1.53 km.

**Table 4:** Latent selection difference (LSD) function comparing caribou use of habitats defined by predation risk (PRED), food availability (FOOD), and proximity to roads (ROAD) across seasons (CALVING: precalving = 0 and postcalving = 1). Main effects estimate relative differences in habitat use across seasons of females without calves, while interaction terms estimate the additional effect of calving (* indicates significance at a = 0.05)

Covariate	β	*SE*	*t*	*p*
Intercept	−1.0339	0.5624	−1.838	.0660
PRED	−7.0889	1.7456	−4.061	<.0001*
FOOD	8.5156	0.5691	14.964	<.0001*
ROAD	1.2331	0.5478	2.251	.0244*
CALVING	6.6243	0.7099	9.331	<.0001*
PRED x CALVING	−19.2338	2.2814	−8.431	<.0001*
FOOD x CALVING	−7.9948	0.6646	−12.029	<.0001*
ROAD x CALVING	−9.8653	0.7183	−13.735	<.0001*

## 4 DISCUSSION

In this study, we tested whether female woodland caribou in Ontario selected local habitats to increase access to high‐energy forages, avoid predation risk posed by wolves, or both. We found that within seasonal ranges, caribou did both, regardless of their reproductive state (Figure 1). We note that both our predation risk and forage availability maps are habitat‐based projections generated by empirically parametrized models (Avgar et al., 2015; Kittle et al. 2015), and whereas we believe they represent a substantial step‐forward compared to traditional habitat selection applications, they may suffer from both inaccuracies and imprecisions. That said, both our study and previous studies using these projections have found consistent and ecologically plausible results (Avgar et al., 2015; McGreer et al., 2015), indicating that these projections are at least highly correlated with the ecological covariates they represent. We also tested whether habitat selection depended on reproductive state such that females with calves at heel selected habitats to offset the increased energetic demands of lactation, to offset the increased vulnerability of their calf to predation, or both. We found that avoidance of predation risk and proximity to roads depended on whether a calf was at heel, but that selection for food availability was not influenced by the presence of young calves.

We found evidence that caribou account for the increased vulnerability of their calf by avoiding predation risk more strongly both within their seasonal range (Figure 1) and within a typical 2.5‐hr displacement of a given location (Figure 2). This observation agrees with similar findings for woodland caribou using different methods and implicit covariates (Bergerud et al., 1984; Pinard et al., 2012). The importance of predation risk in influencing the behavior of reproductive caribou was further supported by our seasonal analysis, where females with a calf at heel increased predation avoidance upon calving to a greater degree than the simultaneous seasonal changes of females that were never observed with calves (Table 4). We can conclude from this that woodland caribou perceive predation risk (whether directly or indirectly) as an important factor of not only their own survival, but the survival of their calf as well. Our findings fit in well with theory previously developed on the ecology of fear (Brown, 1999) and are comparable to the finding that elk sacrifice the use of high‐quality foraging areas in order to avoid predation by wolves (Hernández & Laundré, 2005). This study adds a layer of complexity by suggesting that the landscape of fear depends on reproductive state.

We also considered whether caribou reacted to roads beyond what would be expected by the increased predator density associated with them. We found that the proximity to roads did affect caribou habitat selection within their seasonal range, but that the direction of the effect depended on reproductive state, such that females without a calf selected areas near roads, while those with a calf at heel avoid them (Figure 1; Table 2). It is important to note that this represents the response of caribou to roads after predation risk (which increases near roads and is strongly avoided) has been accounted for. It is possible that caribou with calves perceive roads as a greater threat than caribou without calves or that individual variation in habitat selection and nonrandom calf survival explain the trend. During the calving season, caribou tend to spread out across the landscape, avoiding conspecifics (Bergerud & Page, 1987) and predatory species (Latham et al., 2011). It is likely that they avoid human activity in much the same way. On the other hand, caribou without calves may be less risk‐averse and/or benefit from a factor that is associated with roads. For example, roads in the boreal forest tend to be built across relatively dry, even terrain, and proximity to them may therefore be selected by caribou without calves for ease of movement through the adjacent stands.

Conversely, we did not find that selection for food availability during the calving season depended on whether caribou had a calf at heel (Tables 2 and 3). While we found no evidence that caribou account for the increased energetic demands of lactation through habitat selection, our study is limited to one behavior (habitat selection) during one season (while calves are young). Other behaviors can also influence food acquisition, such as time budgets associated with the functional behaviors of feeding and vigilance (Brown, 1999). For example, both the presence of predators (Laundré, Hernández, & Altendorf, 2001) and human disturbance (Ciuti et al., 2012) have been shown to increase the vigilance of elk (*Cervus canadensis*). It is possible that by avoiding predation risk and human disturbance through habitat selection, females with calves are able to devote more time to foraging. Further, early calf survival is only one component of successful reproduction; successful pregnancy and successful calving are also relevant. We found that although selection for food availability did not depend on reproductive state after calving, females with calves did not adjust selection for food availability upon calving, while females without calves increased selection for food availability across the same season (Table 4). The most likely explanation for this is that females that successfully calved were selecting more strongly for food availability before calving. If so, it could be either that selection for food availability depends on successful pregnancy or that successful calving depends on selection for food availability during pregnancy.

Comparing habitat selection across reproductive state has been attempted before in the woodland caribou system, but no difference in habitat selection between reproductive states was found, likely due to the difficulty of accurately identifying reproductive state in the field (Rettie & Messier, 2000). As a qualitative observation, we found that identifying the calving date of woodland caribou using video collars was likely very accurate. The first recording of each calf typically provided evidence that the calf had just been born that morning, including damp fur, persistent grooming from the mother, the inability of the calf to stand without considerable effort, and in one case even the consumption of birth tissues by the mother. However, calf mortality was less clear. Toward the end of the summer, it was normal to have several days of video recordings between calf sightings, suggesting that the last time the calf was recorded may not necessarily indicate calf mortality on that day. It is possible that combining video observations with other signals that likely depend on the presence of calves, such as movement patterns (Rettie & Messier, 1998), might improve accuracy in estimates of the time of calf mortality.

The ultimate driver of habitat selection patterns is improved fitness, including the odds of both parent and offspring survival. Animals need to rely on proximate cues to differentiate and select habitats (Orians & Wittenberger, 1991), and these cues are not always adaptive (Schlaepfer, Runge, & Sherman, 2002). Our finding that caribou select high‐forage areas and avoid areas with high predation risk suggests that the cues they use to navigate are at least somewhat related to the underlying top‐down and bottom‐up factors that ultimately influence their fitness. Given that upon calving, caribou respond more strongly to predation risk than food availability, it is possible that the availability of these low‐predator areas is the primary constraint on calf survival. This would be true under the condition that caribou are perceiving and responding to predation risk and food availability in an adaptive manner. Even if caribou habitat selection is adaptive, the absolute predation risk and variability of it determine whether the behavior can successfully sustain the population.

Our study was limited to observations from nine individual caribou. Although relatively detailed information was available for each individual, the small sample size does constrain the generality of our results. There are also other environmental factors that could affect differences in habitat selection across reproductive state. Examples of particular relevance to our study area include the gradient in human disturbance (Appendix S1B) and the distribution of other predators (such as black bear, *Ursus americanus*; Pinard et al., 2012), and other ungulates (such as moose; James et al., 2004). The opportunity to address these other factors will depend on further data collection.

## 5 CONCLUSION

We aimed to determine whether the presence of young calves affects the distribution of woodland caribou, identify which top‐down and bottom‐up influences on fitness might drive differences in habitat selection, and provide an example of the utility of video collars in wildlife ecology. We found that habitat selection, and hence the distribution of animals, can depend on reproductive state (the presence and absence of young offspring). We found evidence that for woodland caribou, part of the difference in habitat selection is explained by predation risk and proximity to roads, as females with calves avoid both more strongly than those without to account for the increased vulnerability of their calves. Finally, we demonstrated that there is potential to apply video collars on large terrestrial mammals to identify the reproductive state of individuals.

## CONFLICT OF INTERESTS

None declared.

## AUTHORS' CONTRIBUTIONS

RV conceived the study, compiled the data, carried out the statistical analysis, and drafted the manuscript and figures; TA developed the food availability model and revised the manuscript; GSB and BRP developed the research program; DEB, ARR, JS, and IDT developed the research program and provided edits; and JMF developed the research program, helped conceive the study, and revised the manuscript.

## ETHICS

All procedures adhered to the Ontario Ministry of Natural Resources and Forestry animal care protocols (11‐183 and 12‐183).
