# Supplementary material for: Woodland caribou habitat selection patterns in relation to predation risk and forage abundance depend on reproductive state
Source: Ecol Evol. 2018 May 4;8(11):5863–72. doi: 10.1002/ece3.4124 (PMC6010817; doi:10.1002/ece3.4124)
Supplement: Supplementary file 1 [file ECE3-8-5863-s001.docx]

**APPENDIX A**

To determine whether caribou habitat selection depends on reproductive state at the seasonal-range scale, we fit a resource selection function (RSF) of the used-available design described by Manly et al. (2002). To determine whether caribou habitat selection depends on reproductive state when movement constraints are accounted for, we fit a step selection function (SSF) of the used-available design described by Fortin *et al.* (2005). To determine if females of a given reproductive state selected or avoided any of the landscape covariates, a RSF and SSF was run for each reproductive state (with and without calves at heel) separately. Below are the full results of the four models that correspond with the RSF (Fig 1) and SSF (Fig2) results in the text. RSF coefficients for food availability (dietary digestible biomass), predation risk (relative wolf density), and proximity to roads were estimated using mixed-effects logistic regression. SSF coefficients for the same three covariates were estimated using case-controlled logistic regression.

RSF with calves

| **Covariate** | **β** | **SE** | $\boldsymbol{t}$ | $\boldsymbol{P}$ |
| --- | --- | --- | --- | --- |
| Intercept | -0.2179 | 0.1497 | -1.456 | 0.145 |
| PRED | -14.2386 | 0.4837 | -29.435 | < 0.0001* |
| FOOD | 3.8970 | 0.1651 | 23.605 | < 0.0001* |
| ROAD | -2.1310 | 0.2189 | -9.734 | < 0.0001* |

RSF without calves

| **Covariate** | **β** | **SE** | $\boldsymbol{t}$ | $\boldsymbol{P}$ |
| --- | --- | --- | --- | --- |
| Intercept | -1.5957 | 0.08645 | -18.46 | < 0.0001* |
| PRED | -8.3148 | 0.4363 | -19.06 | < 0.0001* |
| FOOD | 3.2000 | 0.1733 | 18.46 | < 0.0001* |
| ROAD | 1.2990 | 0.1872 | 6.94 | < 0.0001* |

SSF with calves

| **Covariate** | **β** | **SE** | $\boldsymbol{t}$ | $\boldsymbol{P}$ |
| --- | --- | --- | --- | --- |
| PRED | -8.7321 | 1.0745 | -8.127 | < 0.0001* |
| FOOD | 0.6281 | 0.3310 | 1.898 | 0.0577 |
| ROAD | -0.7572 | 0.6714 | -1.128 | 0.2594 |

SSF without calves

| **Covariate** | **β** | **SE** | $\boldsymbol{t}$ | $\boldsymbol{P}$ |
| --- | --- | --- | --- | --- |
| PRED | -3.2617 | 1.0685 | -3.052 | 0.00227* |
| FOOD | 0.9553 | 0.3262 | 2.929 | 0.00340* |
| ROAD | -1.4811 | 0.8784 | -1.686 | 0.09177 |
