# Supplementary material for: Woodland caribou habitat selection patterns in relation to predation risk and forage abundance depend on reproductive state
Source: Ecol Evol. 2018 May 4;8(11):5863–72. doi: 10.1002/ece3.4124 (PMC6010817; doi:10.1002/ece3.4124)
Supplement: Supplementary file 2 [file ECE3-8-5863-s002.docx]

**APPENDIX B**

Because we had individuals from two ends of the study area with known differences in human disturbance, we ran the RSF and SSF models with interaction separately for each end of the study area. Each of these models were based on few individuals (5 in Nakina and 4 in Pickle Lake), so while we are reluctant to draw conclusions from them, we did use them to check the consistency of our results across the study area. All coefficients were estimated using the lme4 package for R 3.0.1. The levels of human disturbance were relatively low in the northwestern end of the study landscape (centered on the township of Pickle Lake) because commercial forestry operations were not permitted, and relatively high in the southeastern end of the study landscape (centered on the township of Nakina) due to commercial timber harvesting. Pickle Lake accordingly has a higher proportion of old conifer stands and lower moose, wolf, and road densities compared to Nakina, where mixedwood and deciduous stands are more common (Mallon *et al.* 2016).

RSF Pickle Lake

| **Covariate** | **β** | **SE** | $\boldsymbol{z}$ | $\boldsymbol{P}$ |
| --- | --- | --- | --- | --- |
| Intercept | -3.4713 | 0.3548 | -9.783 | < 0.0001* |
| PRED | -8.2851 | 0.8187 | -10.119 | < 0.0001* |
| FOOD | 1.5961 | 0.2245 | 7.108 | < 0.0001* |
| ROAD | 49.8726 | 2.0459 | 24.377 | < 0.0001* |
| CALF | 1.1614 | 0.2237 | 5.193 | < 0.0001* |
| PRED x CALF | -2.7568 | 1.0400 | -2.651 | 0.00803* |
| FOOD x CALF | 1.0010 | 0.3207 | 3.121 | 0.00180* |
| ROAD x CALF | -16.8725 | 2.1843 | -7.724 | < 0.0001* |
|  |  |  |  |  |

RSF Nakina

| **Covariate** | **β** | **SE** | $\boldsymbol{z}$ | $\boldsymbol{P}$ |
| --- | --- | --- | --- | --- |
| Intercept | -2.3034 | 0.1719 | -13.399 | < 0.0001* |
| PRED | -10.0359 | 0.5649 | -17.766 | < 0.0001* |
| FOOD | 8.2721 | 0.3718 | 22.248 | < 0.0001* |
| ROAD | 0.6587 | 0.2444 | 2.695 | 0.00704* |
| CALF | 2.4368 | 0.1956 | 12.460 | < 0.0001* |
| PRED x CALF | -7.0141 | 0.8474 | -8.278 | < 0.0001* |
| FOOD x CALF | -3.0418 | 0.4454 | -6.829 | < 0.0001* |
| ROAD x CALF | -2.7780 | 0.3303 | -8.411 | < 0.0001* |
|  |  |  |  |  |

SSF Pickle Lake

| **Covariate** | **β** | **SE** | $\boldsymbol{z}$ | $\boldsymbol{P}$ |
| --- | --- | --- | --- | --- |
| PRED | -6.519 | 1.658 | -3.931 | < 0.0001* |
| FOOD | 0.6661 | 0.4025 | 1.655 | 0.0980 |
| ROAD | -22.75 | 17.41 | -1.307 | 0.1913 |
| PRED x CALF | -13.53 | 3.155 | -4.288 | < 0.0001* |
| FOOD x CALF | -1.649 | 0.7424 | -2.221 | 0.0263* |
| ROAD x CALF | 27.88 | 29.69 | 0.939 | 0.3476 |

SSF Nakina

| **Covariate** | **β** | **SE** | $\boldsymbol{t}$ | $\boldsymbol{P}$ |
| --- | --- | --- | --- | --- |
| PRED | -1.114252 | 1.412841 | -0.789 | 0.43031 |
| FOOD | 1.594950 | 0.555352 | 2.872 | 0.00408* |
| ROAD | -1.621218 | 0.870641 | -1.862 | 0.06259 |
| PRED x CALF | -4.898611 | 1.847658 | -2.651 | 0.00802* |
| FOOD x CALF | -0.236475 | 0.683969 | -0.346 | 0.72954 |
| ROAD x CALF | 0.461733 | 1.093883 | 0.422 | 0.67295 |
