# Supplementary material for: Woodland caribou habitat selection patterns in relation to predation risk and forage abundance depend on reproductive state
Source: Ecol Evol. 2018 May 4;8(11):5863–72. doi: 10.1002/ece3.4124 (PMC6010817; doi:10.1002/ece3.4124)

**APPENDIX C**

**Fig 3:** Empirical step length and turn angle distributions from the trajectories of nine woodland caribou. Locations were recorded by GPS collars between May 15—September 1 of 2011 and 2012 at a fix rate of 2.5 hours.


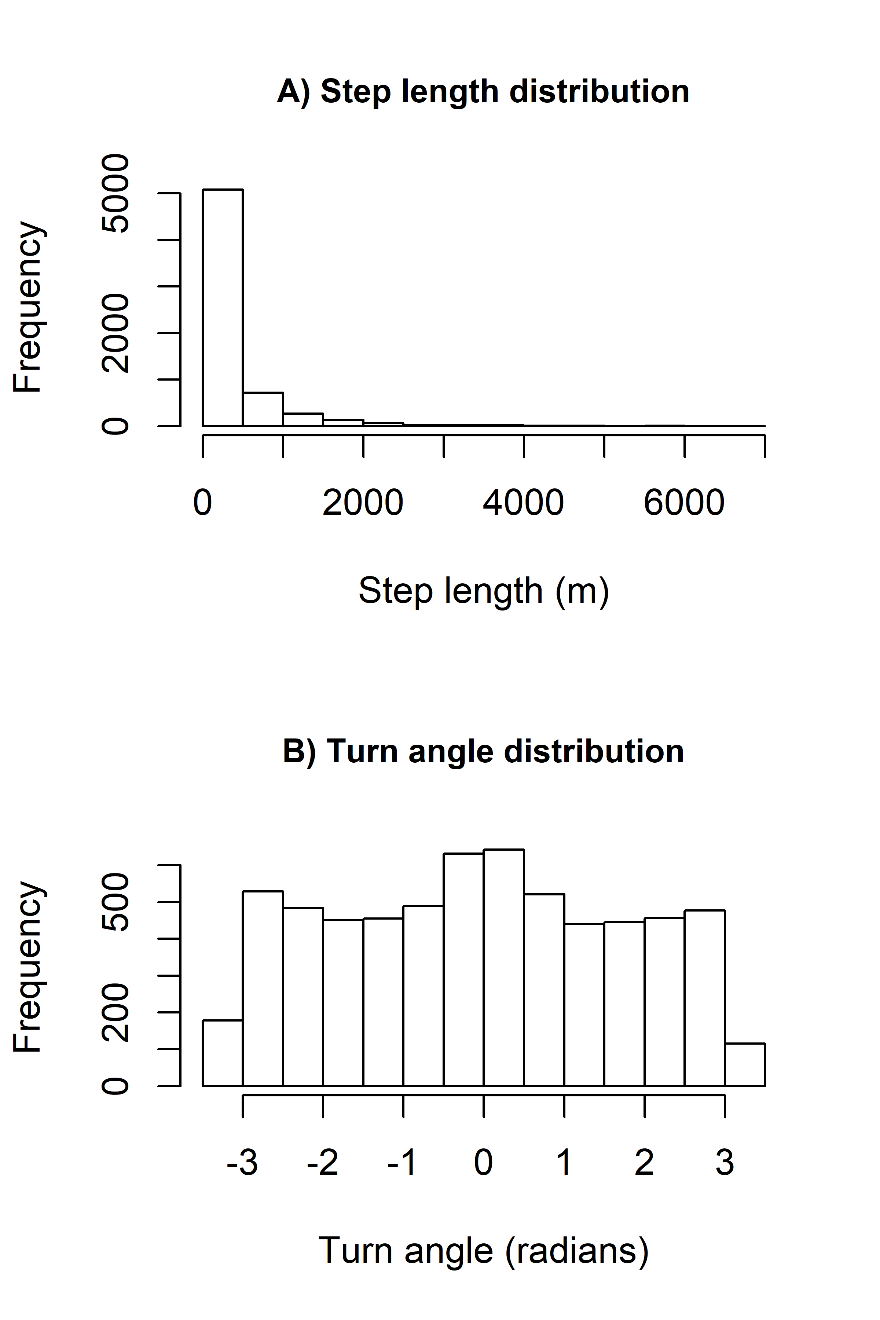

Supplement: Supplementary file 3 [file ECE3-8-5863-s003.docx]
