# Supplementary material for: Woodland caribou habitat selection patterns in relation to predation risk and forage abundance depend on reproductive state
Source: Ecol Evol. 2018 May 4;8(11):5863–72. doi: 10.1002/ece3.4124 (PMC6010817; doi:10.1002/ece3.4124)
Supplement: Supplementary file 4 [file ECE3-8-5863-s004.docx]

**APPENDIX D**

To compare habitat selection of caribou during the pre-calving and post-calving seasons, we used mixed-effect logistic regression to estimate the coefficients of a latent selection difference (LSD) function (Fischer & Gates 2005; Roever, Boyce & Stenhouse 2008). Though relative change in habitat use is directly measured, inference can be extended to relative change in habitat selection if it is reasonable to assume that the same habitats are available across both seasons. We estimated a 95% MCP home range for the pre- and post- calving seasons of each individual to visually assess whether these areas were proximate enough to assume that the pre-calving areas remained available to the animals in the post-calving season. Of the nine individuals, eight had overlapping 95% MCP home range estimations in the pre- and post-calving seasons (Fig 4). The minimum distance between the two seasonal ranges for the individual without overlap (CPL320) was 1.53 km.


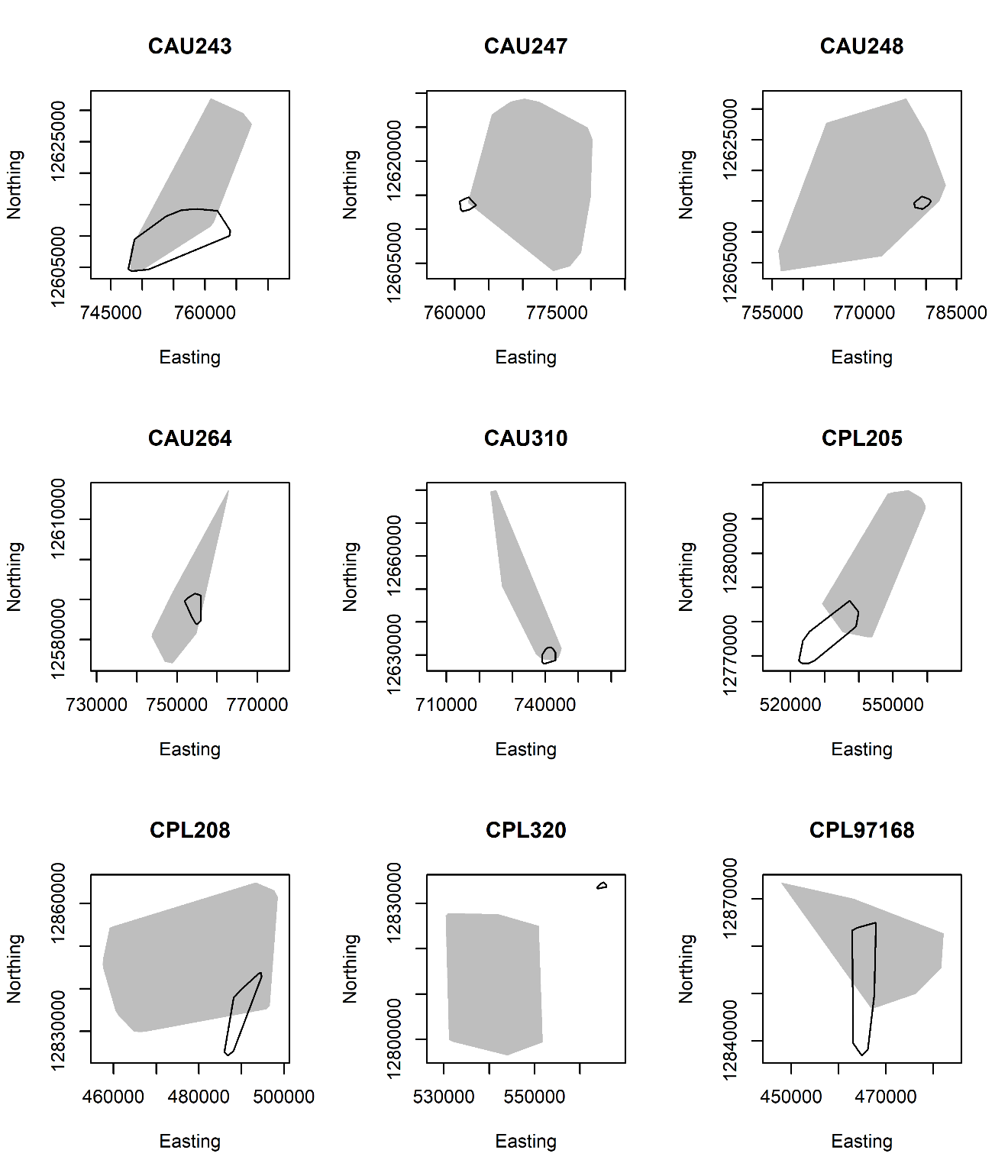


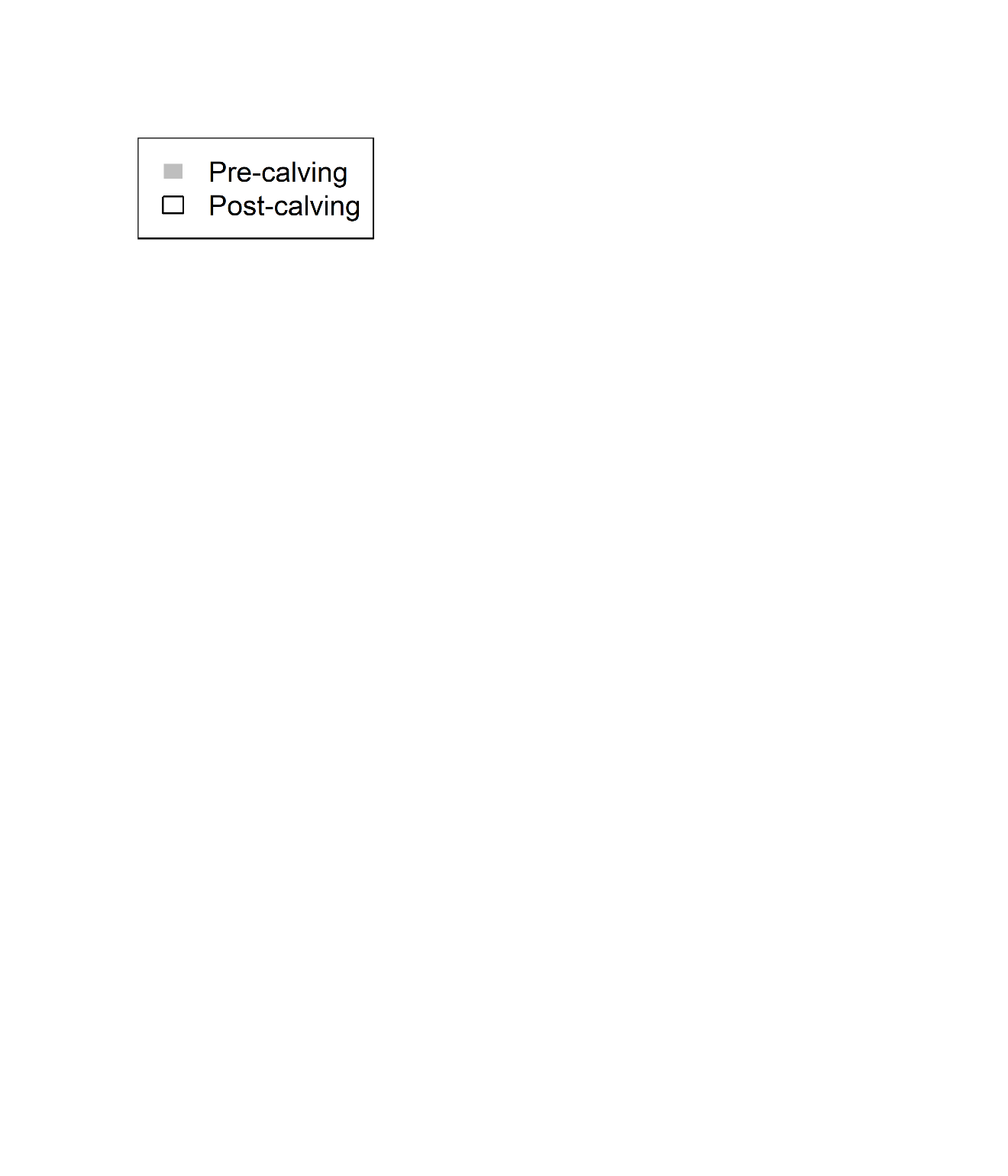


**Fig 4:** 95% minimum convex polygon estimations of pre-calving (grey fill) and post-calving (black border) seasonal ranges of nine individual caribou. These ranges were defined from locations at a 2.5 hour fix rate 40 days before and after calving (for individuals that successfully calved) and 40 days before and after the median calving date (for individuals that did not successfully calf).
